# Looking Beyond the Large Scale Effects of Global Change: Local Phenologies Can Result in Critical Heterogeneity in the Pine Processionary Moth

**DOI:** 10.3389/fphys.2015.00334

**Published:** 2015-11-17

**Authors:** Christelle Robinet, Mathieu Laparie, Jérôme Rousselet

**Affiliations:** Institut National de la Recherche Agronomique, UR633 Zoologie ForestièreOrléans, France

**Keywords:** phenology, climate change, climate warming, insect, *Thaumetopoea pityocampa*, range expansion, species distribution

## Species response to climate change: Why phenology and distribution can be interrelated

Global temperature has increased by 0.85°C between 1880 and 2012, with an acceleration during the last decades and many extreme weather events since about 1950 (IPCC, 2014). A consequence already observed is the change of species distributions. Thermal requirements and constraints are key factors in shaping realized distributions, and climate warming may move or remove these distributional barriers (Walther et al., 2002; Chen et al., 2011). Another consequence of climate warming is a phenology shift for many species (Parmesan, 2006). In plants and ectotherms, shifts in lifecycle timing can result from the direct relationship between temperature and developmental rate (Liu et al., 1995; Chuine et al., 2003). However, phenology is likely driven by a combination of both short-term plastic responses and long-term evolutionary responses to environmental variation (Chuine, 2010; Briscoe et al., 2012).

Consequences of climate change on phenology have often been documented by the study of single life stages (Briscoe et al., 2012). Altered lifecycle timing not only changes climatic conditions undergone by a given stage but can also have cascading effects on subsequent stages. Phenological responses to climate change are thus limited by tradeoffs across the lifecycle, as stages are not independent and often have contrasting thermal sensitivities and requirements (Briscoe et al., 2012). Phenology shifts have been largely studied in plants, showing the role of temporal optimization to cope with several stage-specific constraints (Chuine, 2010). In insects, the overall phenological answer depends on similar complex interactions among life stages with antagonistic or non-additive effects (Briscoe et al., 2012). In turn, these tradeoffs necessarily differ across the distribution due to spatial heterogeneity in climatic conditions and inter-population differentiation. Yet, the relationship between phenology and range shifts has rarely been investigated. Its study requires long time-series of phenology and large-scale surveys of distribution over time, but such historical datasets are rare.

The pine processionary moth (PPM), *Thaumetopoea pityocampa*, is a well-documented Lepidopteran insect which extends its distribution northwards and to higher elevations in Europe as a response to attenuated cold constraint since the 1990s (Roques et al., 2015). This range expansion is acknowledged to be one of the few responses to climate change with direct causal relationship demonstrated (Rosenzweig et al., 2007). For this species, historical data are available not only on distribution at continental scale, but also on phenology and its variation within and among regions (Géri, 1980; Abgrall, 2001; Roques, 2015). Here, we discuss how climate change could alter phenology in this emblematic species, and how climate spatial heterogeneity interacts with phenology, making the mechanism of range expansion more complex than initially thought.

## Pine processionary moth, an insect under multiple thermal constraints

PPM is a capital breeder achieving one generation per year (except in case of extended diapause), with winter larval development (Battisti et al., 2015). Eggs are laid in summer and hatching begins approximately 1 month later. Larvae gregariously build a silk tent in pine trees, continue development during winter, and make a procession from their host tree to pupate in the soil in late winter or early spring. After a pupal diapause with flexible duration, adults emerge in summer. Most emergences occur on a short duration (flight peaks) regarding the whole flight period. Because of a short adult lifespan, the mating success depends on synchronous emergences (Démolin, 1969a).

Following Huchon and Démolin (1970), phenology variability is mainly limited by three stage-specific constraints: (a) first instar larvae (L1) are vulnerable to high summer temperatures, (b) second instar larvae (L2) are vulnerable to first autumnal frosts, (c) late instars larvae (L3-L5) are vulnerable to minimal temperatures during autumn and winter, a factor identified as limiting PPM range expansion (Battisti et al., 2005).

**Figure 1:**
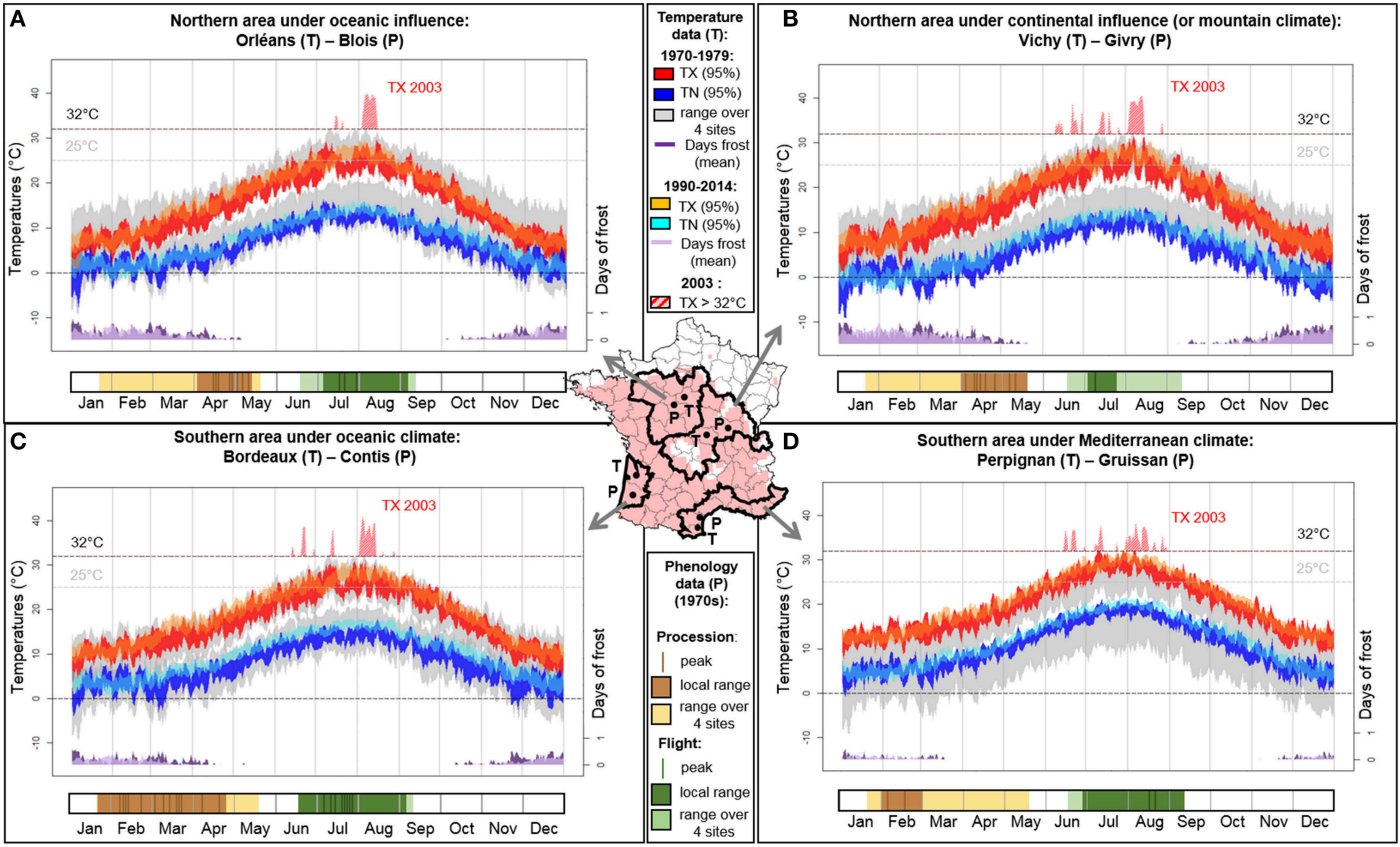
**Climate (upper part of each panel; data from http://eca.knmi.nl, Klein Tank et al., 2002) and PPM phenology (procession and flight dates; bottom part of each panel; derived from Abgrall, 2001) in four bioclimatic regions in France**. **(A)** northern area under oceanic influence, **(B)** northern area under continental influence (or mountain climate), **(C)** southern area under oceanic influence, and **(D)** southern area under Mediterranean climate. Hatching occurs 1 month after flights, and L1, L2, and later larval instars until pupation procession are respectively constrained by maximal temperatures in summer, first frosts, and minimal temperatures in winter. Late flight prevents exposure of the L1 progeny to high temperatures (Mediterranean climate) while early flight prevents exposure to early cold snaps (climate with continental influence). Pupation processions are late when winters are cold because of slowed larval development (northern areas) and early when winters are mild (southern areas). Under lower climate constraints (south-western area), flight and procession dates are more widely distributed over time. With climate warming and more frequent heatwaves, PPM phenology may change and its overall distributional response remains difficult to predict despite current beneficial effects on winter larval survival. Daily temperatures (95% confidence interval for TX, daily maximal temperatures; TN, daily minimal temperatures) and mean number of days of frost are shown for 1970–1979 and 1990–2014 (corresponding to the PPM range expansion period; Robinet et al., 2014). Biological thresholds of 0, 25, and 32°C are given for reference. Maximal temperature overreaching 32°C during the 2003 heatwave is represented in red crosshatched. The map gives the PPM distribution in winter 2010–2011 (in light red) in France and the location of the sites where phenology (P) was recorded and where temperature data (T) were retrieved within each of the four bioclimatic zones (see Boutte, 2014, for the definition of these bioclimatic zones).

PPM phenology was recorded in the 1970s in various French regions (Abgrall, 2001) by monitoring pupation processions and adult flights. Additionally to elevation, latitudinal (South/North) and oceanic-continental (West/East) gradients shape climate variability in France. Here we consider four regions with differentiated PPM phenologies (Abgrall, 2001) and representative of the main French climates (Boutte, 2014; Figure 1).

## How spatial heterogeneity in thermal constraints may drive local phenologies

### (a) First instar larvae are vulnerable to high summer temperatures

Following Démolin (1969b), temperatures above 32°C could perturb physiological processes in eggs and/or larvae, and cause epizootics. He considered that these effects would occur where monthly mean of maximal temperatures are above 25°C. Recent studies showed that eggs from a northern French population are not negatively affected by such temperatures (Robinet et al., 2013), while survival of L1 and L2 from Portuguese populations are negatively affected by temperatures of 36°C and 40°C, respectively (Santos et al., 2011). In France, such high temperatures are the most likely under Mediterranean climate (Figure 1D). However, in this area, heat-vulnerable larvae mostly hatch after the highest summer temperatures because flight peaks are late (Figure 1D), and can thus experience more suitable temperatures (never reaching 32°C after late August; Démolin, 1969b).

### (b) Second instar larvae are vulnerable to first autumnal frosts

According to Huchon and Démolin (1970), early instar larvae may be particularly cold-sensitive and must hatch early enough to allow molting into at least the third instar to better resist first cold snaps. In France, early frosts (September) are the most likely in the north-eastern area (Figure 1B) but larvae can reach the third instar before the frosts as a consequence of early flight peaks (early July). Flight peaks occur later (mid-late July) in north-western area but cold snaps also appear later (October) due to oceanic influence (Figure 1A). The absence of early cold snaps before November in Mediterranean climate allows the development of larvae even following late flights (Figure 1D).

### (c) Late instars larvae are vulnerable to minimal temperatures during autumn and winter

Huchon and Démolin (1970) reported the role of minimal temperatures in January-February associated with solar radiation on PPM distribution and lifecycle. Different lethal temperature thresholds have been reported (less than −12°C, Huchon and Démolin, 1970; −17°C, Hoch et al., 2009) but all appear low enough to withstand usual winters in the areas considered. Instead, the main detrimental effects of cold are chill injury due to cumulative negative temperatures (Hoch et al., 2009) and inability to reach the foraging thresholds (Battisti et al., 2005). Cold temperatures in northern areas delay the beginning of processions (Figures 1A,B) whereas milder winters in southern areas allow faster larval development and earlier processions (Figures 1C,D).

### (d) Larval vulnerabilities may determine adult flight timing

Factors triggering the end of pupal diapause, and thus adult emergences and local synchrony, are unknown. Nevertheless, the duration of pupal diapause cannot be a simple by product of pupal procession time and climate experienced by the pupae. Indeed, historical data show lower variability for flight periods than procession periods within regions (Abgrall, 2001; Figure 1). Moreover, the pupal diapause lasts longer in the South than in the North, despite earlier pupations and warmer climates (Roques and Battisti, 2015; Figure 1). This contrasts with typical relationship between temperature and growth rate in ectotherms, thereby suggesting a synchronizing mechanism. To date, there is no evidence that PPM adults are subjected to direct climatic constraints either. We suppose that adult timing is instead indirectly constrained by the aforementioned vulnerabilities of the offspring: hot summers promote late flight peaks and delayed hatching, whereas cold autumns promote early flight peaks and early hatching.

## How local climates and phenologies lead to heterogeneous answers to climatic anomalies

Extreme weather events can occur with high spatial heterogeneity (Seneviratne et al., 2012), and increased temperature fluctuations can affect species fitness (Williams et al., 2012). In France, maximal temperatures during the 2003 summer heatwave were 8–13°C higher than the mean over 1990–2014 (Figures 1A–D). While French populations collapsed in the two northern areas following this heatwave (Bouhot-Delduc, 2005), an unprecedented altitudinal expansion was observed in the Italian Alps due to unusually numerous nights above the flight threshold (14°C) during this summer, elicitating female flight (Battisti et al., 2006). Although, early-summer flight peaks in these three regions led to hatching during the thermal peaks, L1 experienced suitable temperatures in the Italian Alps (rarely reaching 32°C, Andrea Battisti, Pers. Com.) while they faced lethal conditions in northern French areas (40°C, Figures 1A–B).

Another component of heterogeneous responses to climate anomalies is differentiated phenologies. Despite similarly high temperatures, French populations did not crash in areas where late flight peaks occur (Figure 1C) or prevail (Figure 1D), thus allowing hatching of vulnerable L1 after the constraint. This heatwave illustrates how the fate of local populations can be triggered by (1) spatial heterogenity of large-scale climatic events, but also (2) phenology and differential thermal sensitivity among life stages and populations.

## Discussion

Climate change between 1970–1979 and 1990–2014 resulted in an average winter warming in the PPM bioclimatic zones considered (October to March: +0.8 to +1.5°C; Figure 1), easing the reach of foraging threshold (Battisti et al., 2005; Robinet et al., 2007). These beneficial effects facilitating range expansion across Europe have focused attention on late instars larvae, while constraints on other life stages have been overlooked. Yet, the increasing frequency of autumnal heatwaves (Luterbacher et al., 2007) may release the constraint of early frosts on second instar larvae. Warmer summer nights are also expected to accelerate range expansion where female take-off threshold is a limiting factor (Battisti et al., 2006). However, future climate change may also have detrimental effects on the PPM. For instance, the hypothesized prejudicial 25°C threshold is already being reached more frequently in the four areas (+14 to +34 days). Additionally, constraints on winter development of later instars may persist (Robinet et al., 2012) as cold waves may still occur in the 21st century (Kodra and Ganguly, 2014).

Because of cascading phenological effects, the contrasted consequences of climate change on different life stages cannot be considered independent. This may be critical at range margins where populations already face limiting conditions. Range expansion was the slowest in the north-eastern area under continental influence, where warm summers (3a) and early cold snaps (3b) tend to apply antagonistic constraints on PPM lifecycle (3d). Moreover, PPM is widespread across Europe, North Africa and Minor Asia, with locally differentiated phenologies, except in continental regions where opposing constraints can be too strong to be overcome by phenological responses. Conversely, high phenological variability in processions and flights in the south-western area suggests less stringent climatic conditions due to milder summers and winters (Figure 1C). Such phenological heterogeneity means that varied life stages experience similar thermal conditions, which in turn alters the survival of colonies in case of extreme climatic events.

Since the early 2000s, an increasing number of erratic PPM phenologies are being reported in France. The most striking illustration is the co-occurrence of both typical (March-May) and atypical (October-January) processions within the northern area under oceanic influence (Boutte, 2012; our own observations in 2006, 2011, and 2014). We expect that the increasing frequency of autumnal heatwaves (Luterbacher et al., 2007) will favor such events of accelerated larval development, but it is unknown whether these phenological discrepancies are viable and maintained over generations. However, in Portugal, a population has durably shifted its phenology toward a summer larval development with higher heat resistance, and co-occurs with typical winter populations (Santos et al., 2011). This illustrates how varied phenologies may broaden the range of climatic conditions the PPM can survive locally, and may ultimately facilitate the persistence or spread to new areas with climate change.

## Conclusion

Climate warming can exert opposing pressures on the distribution of insects, not only among species, but also within species due to contrasted susceptibility of life stages and heterogeneous phenologies, ultimately altering local survival. Predicting insect distribution under climate change is thus challenging as models should consider not only common concepts of species spread, but also stage-specific thermal thresholds and inter-populational heterogeneity in phenology, acclimation and physiological adaptation (Chuine, 2010; Briscoe et al., 2012; Godefroid et al., 2015). Monitoring both distributional and phenological changes is now crucial to develop and feed such models.

## Author contributions

CR and JR prepared the phenology and climate datasets; CR, ML, JR worked on the data and wrote the paper.

## Funding

This study was supported by PCLIM network funded by metaprogram ACCAF of INRA.

### Conflict of interest statement

The authors declare that the research was conducted in the absence of any commercial or financial relationships that could be construed as a potential conflict of interest.
